# Fatigue Performance of RC Beams Strengthened with CFRP under Overloads with a Ladder Spectrum

**DOI:** 10.3390/s18103321

**Published:** 2018-10-03

**Authors:** Zhan-Biao Chen, Pei-Yan Huang, Zheng-Wei Li, Xin-Yan Guo, Chen Zhao, Xiao-Hong Zheng, Yi Yang

**Affiliations:** 1 School of Civil Engineering and Transportation, South China University of Technology, Guangzhou 510640, China; ctryancccz@mail.scut.edu.cn (Z.-B.C.); xyguo@scut.edu.cn (X.-Y.G.); zhaochen@scut.edu.cn (C.Z.); xhzheng@scut.edu.cn (X.-H.Z.); yiyang@scut.edu.cn (Y.Y.); 2 State Key Laboratory of Subtropical Building Science, South China University of Technology, Guangzhou 510640, China; 3 Guangzhou Communication Investment Group CO. LTD., Guangzhou 510288, China; lizhengwei@mail.gjtjt.com

**Keywords:** vehicle overload, overload ladder spectrum, fatigue performance, carbon fiber reinforced polymer (CFRP), reinforced concrete (RC) beams

## Abstract

Vehicle overload is detrimental to bridges and traffic safety. This paper presents a study on the fatigue performance of typical reinforced concrete (RC) beams of highway bridges under vehicle overload. A definition method of vehicle overload and a construction method of overload ladder spectrum were first proposed based on traffic data acquisition, statistical analysis and structural calculation of the highway bridges in Guangzhou. A fatigue experimental method was also proposed with the three-ladder vehicle overload spectrum, and the fatigue tests of 15 RC beams strengthened with carbon fiber reinforced polymer (CFRP) under three loading levels were then carried out. The fatigue performance and the failure mechanism of the strengthened beams were presented and discussed, and two fatigue life prediction methods were proposed with the established modified Palmgren-Miner rule and the loading level equivalent method respectively. The results showed that the fatigue performance of the strengthened RC beams was severely degraded under overload ladder spectrum compared with that under constant amplitude cyclic load, and the life prediction methods were proved effective.

## 1. Introduction

The vehicle over-limit cases are unavoidable in China, though measures have been taken to limit the quantity of cargo transport [1]. The bridges are easily subjected to over design loading if the over-limit cases take place and the bridges work beyond their design load is referred to as overload henceforth. Overload causes damages and defects of bridge structures, which subsequently shortens the service life of bridges and even endangers the safety of transportation [2,3]. However, the mechanism of overload, the damage evolution law and failure modes of bridge structures under overload are not at all clear. On the other hand, strengthening RC members using fiber reinforced polymer (FRP) has become more and more popular [4,5,6,7], which can well improve the fatigue behavior. In addition, it was found that ductile adhesives with greater strength would help improve the fatigue performance by delaying the occurrence of debonding [8]. Therefore, it has been an important issue to investigate the fatigue performance of bridge structures including reinforced concrete (RC) structures strengthened with FRP under overload.

At present, effects of overload on the mechanical properties of RC structures strengthened with carbon fiber reinforced polymers (CFRP) have attracted attention from the academic community. Dawood et al. [9] investigated the load-bearing capacity of RC beams strengthened with CFRP and the results from their study showed that externally bonded high modulus CFRP materials can effectively reduce the residual deflection of the strengthened RC beams. Elrefai et al. [10] carried out several fatigue tests of the overload-damaged RC beams strengthened with prestressed CFRP. The results from Elrefai et al. [10] showed that prestressed CFRP strengthening is an efficient technique to increase the fatigue performance of the overload-damaged RC beams. Sun et al. [3] conducted an experimental study on the influences of vehicle overload on the static and fatigue performance of RC bridge girder externally bonded CFRP laminates. The displacement and crack propagation behavior of RC bridge girder strengthened by CFRP under vehicle overload were preliminarily discussed, and the residual strength of RC bridge girder after cyclic loading was experimentally studied. However, the overloads in the above studies were generally limited to a fixed value, and the fatigue tests of the strengthened components were only the confirmatory tests. Additionally, previous studies fail to clarify the effects of vehicle load spectrum or variable amplitude load, while the fatigue tests of the strengthened components were not conducted systematically.

Actually, highway bridges are served under vehicle random loads. Consequently, it is essential to study vehicle random load spectrum, otherwise the fatigue performance of RC bridge structures strengthened with CFRP under vehicle overload cannot be properly understood [11]. Therefore, researchers [12,13,14] have studied the vehicle load spectrum for a certain type of bridge or the bridges in a certain area respectively. Yang et al. [15] proposed a vehicle load spectrum described by a probability distribution, through conducting a comparative analysis between areas with or without cargo transport limit. Studies on traffic data acquisition, statistical analysis and structural calculation of the national highway G321 and a Guangzhou highway were carried out by the research group of the authors, and the vehicle random load spectrums were proposed [16,17]. The proposed spectrum construction method provides a good foundation for experimental study on fatigue performance of bridge structures under vehicle random loads.

The complex random load spectrum would increase the difficulty of experimental study. It has been simplified to two or three ladders load spectrum in some previous researches [18,19,20]. Hosoi et al. [18] investigated the fatigue characteristics of CFRP laminates under a two-stage variable amplitude cyclic loading (two-ladder load spectrum). The results show that the Palmgren-Miner linear damage rule may not be valid in this case due to the effect of loading sequence. Mohamadi et al. [19] found a relatively larger estimation error (36.8–56.5%) of life prediction using Palmgren-Miner rule with the fatigue testing data of FRP strengthened RC beams under three-ladder load spectrum (three-stage variable amplitude cyclic loading). The results of Found et al. [20] show that the Palmgren-Miner linear damage rule may no longer be valid for woven carbon fiber reinforced laminates under two-stage fatigue loading. These fatigue experiments did not consider the influence of vehicle overload, and the testing data is not enough. To gain in-depth understandings on the fatigue performance of CFRP strengthened RC structures, preliminary fatigue experimental studies under three-ladder overload spectrum were carried out by this research group [21,22]. However, the previous studies fail to provide a proper fatigue experimental method under vehicle overload. Moreover, the fatigue failure mechanism and life prediction method of RC beams strengthened with CFRP under vehicle overload spectrum have not been properly improved.

In view of the above considerations, there is a need to carry out fatigue experiment of RC beams strengthened with CFRP considering vehicle overload. The failure mechanism and life prediction method also need to be improved. Therefore, the definition method of vehicle overload and the construction method of overload ladder spectrum were proposed in this paper, based on traffic data acquisition, statistical analysis and structural calculation of a highway in Guangzhou. A fatigue experimental method of RC beams strengthened with CFRP under three-ladder vehicle overload spectrum was proposed, and the fatigue tests were carried out. The fatigue failure mechanism of RC beams strengthened with CFRP under overload spectrum was presented and discussed, and two life prediction methods were proposed.

## 2. Overload Ladder Spectrum

Since vehicle load is the major live load of highway bridges, it plays an important role in the design of highway bridges. In this paper, one month’s traffic data of a highway in Guangzhou was continuously collected by manual survey, weigh-in-motion (WIM) and vehicle capture sensing system. The definition and calculation method of overload was proposed, through the analysis of the loading effect for the typical bridge structure of this highway—20 m simply supported hollow slab beam, under vehicle random load spectrum. Then, an overload ladder spectrum was generated for variable amplitude fatigue experiments.

### 2.1. Definition of Overload

The upper structures of the bridge mainly bear the flexural moment caused by dead load from its own weight and vehicle live load. $M_{c1}$ represents the critical moment when tensile stress occurs in the lower edge of flexural RC component, and $M_{c2}$ represents the ultimate flexural moment of the RC component considering the safety coefficient K. The minimum value of $M_{c1}$ and $M_{c2}$ is taken as the critical moment to define overload. The moment bigger than the critical moment is the overload moment of the RC component. Moreover, the vehicle load greater than the loading value determined by the overload moment is defined as overload.

For the above 20 m simply supported hollow slab beam, on the one hand, using finite element method, the moment $M_{c1}$ was obtained, $M_{c1}=1300\;\mathrm{kN}\cdot m$. On the other hand, according to the design code [23], the ultimate flexural moment $M_{u}$ was calculated as $M_{u}=2308\;\mathrm{kN}\cdot m$. Besides, the safety coefficient K [23] was:

(1) $$K=\gamma_{0}\gamma_{s}\gamma_{fs},$$

where $\gamma_{0}=1.00\sim 1.10$, $\gamma_{s}=1.326$, $\gamma_{fs}=1.20$ are the structural coefficient, loading coefficient, and material coefficient respectively.

Put these coefficients into Equation (1) then the safety coefficient was obtained. In addition, the moment $M_{c2}$ can be calculated by:

(2) $$M_{c2}=\frac{1}{K}\cdot Mu,$$

According to Equations (1) and (2) and the value of Mu, the ultimate bending moment considering safety factor *K* can be obtained: $M_{c2}=1318\sim 1443\;\mathrm{kN}\cdot m$.

Because $M_{c1}<M_{c2}$, the critical overload moment $M_{o}$ of the hollow slab beam can be determined as: $M_{o}=M_{c1}=1300\;\mathrm{kN}\cdot m$.

### 2.2. Overload Moment of Vehicle Live Load

The critical overload moment of the hollow slab beam, $M_{o}$, includes two parts of moments, one from the bridge dead load and another one from the vehicle live load. Overload moment of vehicle live load needs to be separated out to construct a fatigue experimental spectrum of the vehicle overload. The constant moment $M_{1}$ caused by dead load from its own weight can be calculated with the design data [22], and the result of $M_{1}=860\;\mathrm{kN}\cdot m$. Therefore, critical overload moment $M_{vc}$ caused by vehicle live load can be obtained:

(3) $$M_{vc}=\frac{M_{o}-M_{1}}{\eta },$$

where, η=0.549, is the transverse distribution coefficient and calculated with the method of the literature [23]. That is to say, the vehicle load which caused the moments bigger than $M_{vc}=800\;\mathrm{kN}\cdot m$ was defined as overload for the 20 m hollow slab beam.

**Figure 1:**
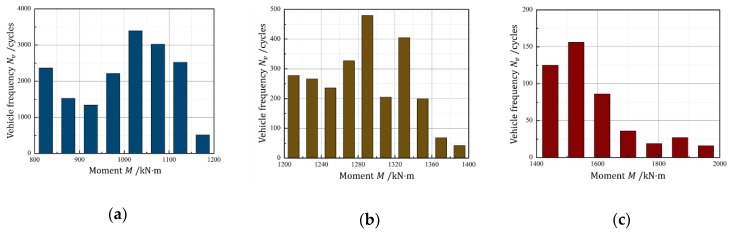
Frequency distributions of vehicle overload moments M: (**a**) [800, 1200] kN·m; (**b**) (1200, 1400) kN·m; (**c**) [1400, 2000) kN·m.

According to the above method, to establish three-ladder overload spectrum by vehicle overloads, the overload moments of vehicle live load bigger than $M_{vc}$ were divided into 3 intervals: [800, 1200] kN·m, (1200, 1400) kN·m and [1400, 2000) kN·m. The frequencies of the overload moments in each interval were shown in Figure 1 from the traffic data.

### 2.3. Vehicle Overload Ladder Spectrum

The moments in three intervals shown in Figure 1 were equivalent to constant amplitude moments respectively, $M_{ve}$, according to linear damage accumulation rule. Considering the transverse distribution coefficient η, equivalent overload moments of the beams under most unfavorable conditions, $M_{vo}$, were obtained, as shown in Table 1. The overload level under the coupling action of $M_{vo}$ and $M_{1}$ and daily vehicle load frequencies of three intervals were also listed in Table 1.

Through static loading test, the ultimate bearing capacity of the specimens used in fatigue experiments was obtained, $P_{u}=45\;\mathrm{kN}$, and ultimate moment $M_{tu}$ of the specimens was calculated accordingly. On the basis of overload level and load frequencies in Table 1 with a stress ratio R=0.1, the maximum load $P_{max}$ and minimum load $P_{min}$ of the three-ladder overload spectrum (experimental spectrum) relative to three intervals were obtained. Then, characteristic values of the experimental spectrum can be listed, as shown in Table 2.

**Figure 2:**
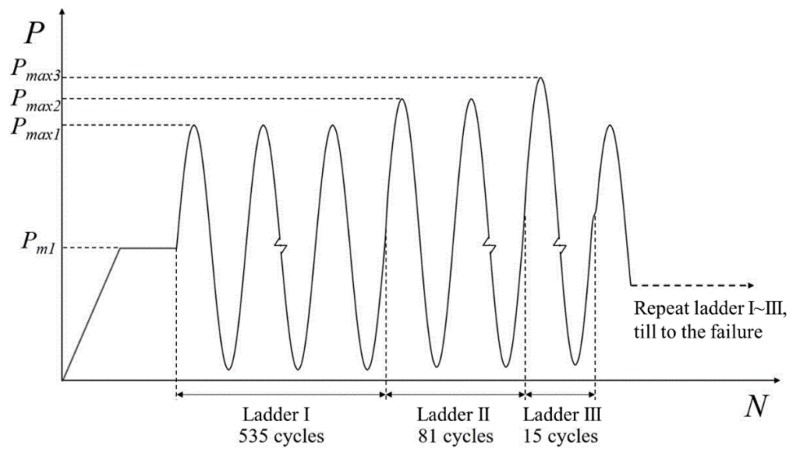
Three-ladder overload testing spectrum.

The characteristic values were imported into overload spectrum construction program. Then, according to the form of sine wave and loading frequency of each ladder, the overload ladder spectrum for variable amplitude fatigue tests was obtained, as shown in Figure 2.

## 3. Fatigue Experiments under Overload Ladder Spectrum

In this paper, fatigue experiments of RC beams strengthened with CFRP under overload ladder spectrum were carried out to obtain fatigue testing data, discuss failure mechanism and analyze fatigue lives of the strengthened beams.

### 3.1. Specimens and Materials

**Figure 3:**
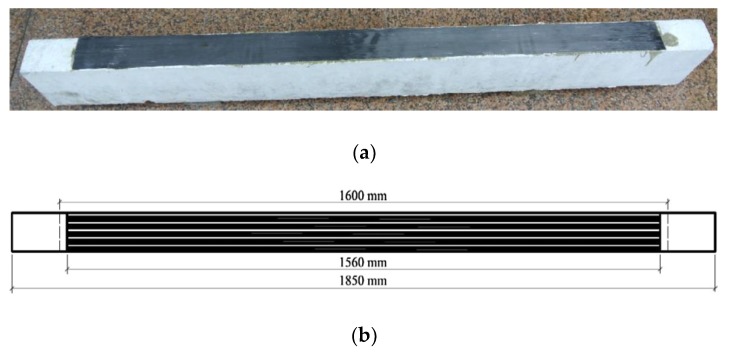
Specimen: (**a**) RC beam strengthened with CFL; (**b**) Size of CFL pasted at the bottom of RC beam.

Three-point bending RC beams strengthened with carbon fiber laminate (CFL) [24] were as the specimens in this study. The size of the RC beam was 1850 mm long × 100 mm wide × 200 mm high with the span length L = 1600 mm. The CFL was pasted on the bottom of the RC beam, as shown in Figure 3. Totally 15 specimens were fabricated.

**Figure 4:**
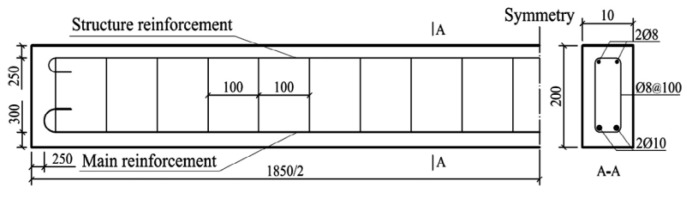
Steel bars used in RC beam.

The strengthened RC beam mainly consisted of concrete, steel bar and CFL. Composition proportion of concrete was *m_c_:m_w_:m_s_:m_a_* = 1.0:0.5:2.06:3.66 (cement: water: sand: gravel). Mechanical properties of the concrete are shown in Table 3, according to *Test Methods of Cement and Concrete for Highway Engineering* (JTG E30-2005) [25]. The main steel bars were Grade II Φ10 and the other steel bars were Grade I Φ8. The reinforcement ratio was 0.981%, as shown in Figure 4. The elastic modulus, Poisson’s ratio, and yield stress of the main steel bar were *E_s_* = 206 GPa, *ν_s_* = 0.3 and *f_y_* = 307 MPa, respectively. CFL (pre-immersion laminate) was 1560 mm long, 100 mm wide, 0.23 mm thick and was weaved with *T700-12k* carbon fiber silk made in TORAY Co., Tokyo, Japan, and immersed with epoxy resin by this research group. Basic mechanical properties of CFL are shown in Table 4. Adhesive used between concrete and CFL was A and B epoxy adhesive produced in Shenliling Co., Changsha, China. The thickness of A and B epoxy adhesive was totally about 0.2 mm, and most of the adhesive penetrated into the concrete. The shear strength of A and B epoxy adhesive was 14 MPa, and its working temperature was −30~+100 °C.

### 3.2. Experimental Method

**Figure 5:**
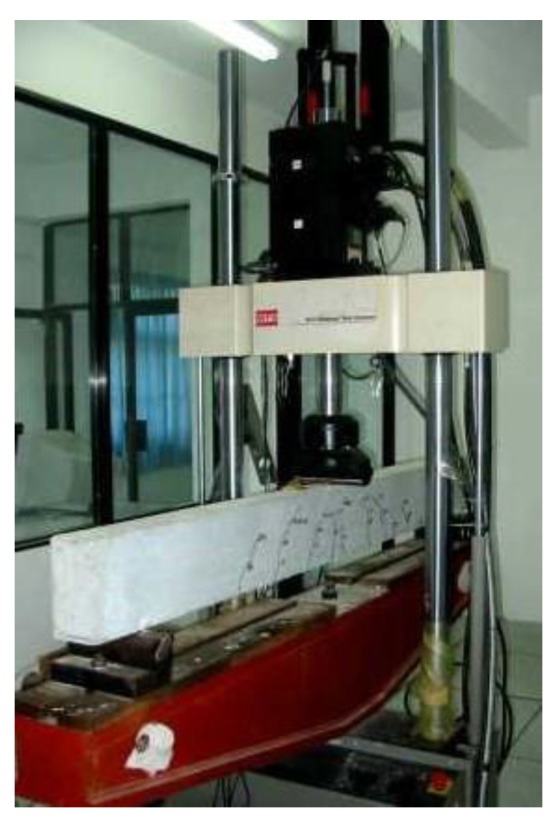
Testing System.

The experiments were carried out on the modified *MTS810* test system, and supporting platform was made by our research group, as shown in Figure 5. The overload ladder spectrum was imported into controller of *MTS810* (Eden Prairie, MN, USA) and loading curve was shown in Figure 2. Specimens were tested till failure. Three-point bending was used with force control mode and stress ratio was R=0.1, and loading frequency was 8 Hz.

15 specimens were divided into 3 groups. There were 5 RC beams strengthened with CFL in each group. In addition, considering the deformation of steel bars and analysis of fatigue lives, loading levels of group A1 were the same with the three-ladder overload spectrum as shown in Table 2. Loading amplitude of group A2 was 10% larger than A1, and that of group A3 was 15% larger than A1. The maximum and minimum loads, mid-span deflections of the specimens during the experiment were recorded. The MTS810 testing system automatically recorded loads, mid-span deflections of the beam and load cycles, where 10 sets of data were recorded during each loading cycle. Detail experimental conditions and results are shown in Table 5.

## 4. Failure Mechanism of the Strengthened Beams

### 4.1. Stiffness Degradation

**Figure 6:**
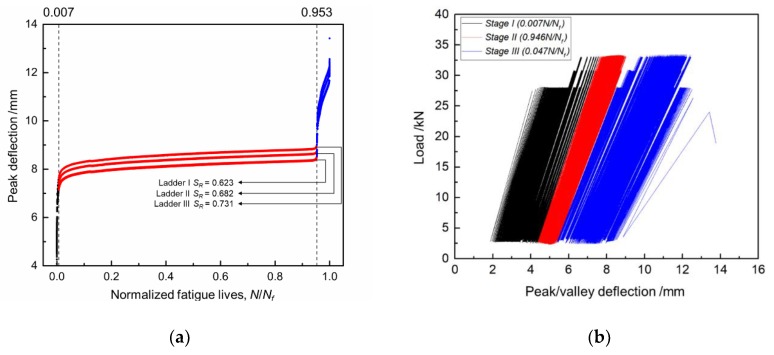
Mid-span deflection curves of specimen No. A13: (**a**) Peak deflection~$N/N_{f}$ curves; (**b**) Load~Peak/valley deflection curves.

The mid-span deflection curves of specimen No. A13 under ladder overload spectrum were shown in Figure 6. The mid-span deflection curves of other specimens were similar. It can be seen that the mid-span deflection of strengthened beam presented a three-stage growth pattern.

At stage I ($N/N_{f}\leq 0.007$), the beginning of fatigue test, as shown in the black curves of Figure 6, the mid-span deflection grew rapidly under every overload ladder. The reason was the rapid degradation of strengthened beam’s stiffness, which was caused by crack initiation and quick propagation on concrete.

After a short time of rapid growth, the mid-span deflection of strengthened entered a steady growing stage: stage II ($0.007\leq N/N_{f}<0.953$). In this stage, the mid-span deflections of three overload ladders were different, but all of them grew slowly under similar rate, which can be seen at the red curves of Figure 6a. In stage II, main crack in concrete propagated slowly while the deformations of main steel bars and CFL were small. All of these led to a slowly stiffness degradation rate of the strengthened beam.

At the end of steady growing stage, the mid-span deflection entered an unstable growing stage: stage III ($N/N_{f}\geq 0.953$), the mid-span deflection grows rapidly under all of three overload ladders, as shown in the blue curves of Figure 6. In this stage, stiffness degraded rapidly till to the failure of the strengthened beam. This was a comprehensive result of yielding and final fracture of the main steel bars, quick propagation of the main crack, propagation of interface crack and crush of concrete.

### 4.2. Failure Process and Failure Mode

Corresponding to the stiffness degradation process (the change process of deflection curve) of the strengthened beam, the fatigue failure process of the RC beam strengthened with CFL under overload ladder spectrum was mainly accompanied by the following phenomena: main crack initiation and propagation on concrete, interfacial crack initiation and propagation in CFL-concrete interface (CFL debonding), fatigue damage accumulation and yielding of main steel bars, and crushing of concrete in compression area.

**Figure 7:**
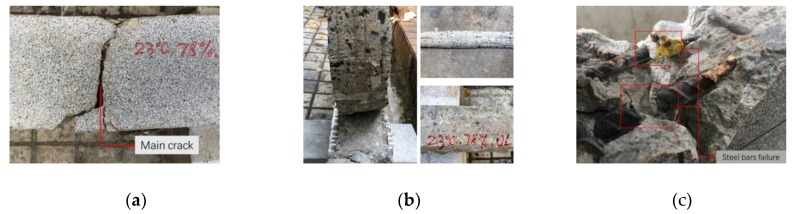
Fatigue failure process of the strengthened beams: (**a**) Main crack in concrete; (**b**) CFL debonding; (**c**) The main steel bars failure.

1. Initiation and propagation of main crack. At the beginning of the fatigue test, when the load was small, there were cracks initiation in concrete. In stage I, one or two macro cracks generated in lower edge of mid-span concrete, and one crack quickly grew into main crack which would result in fatigue failure of the specimen, as shown in Figure 7a. As the fatigue loads continued to operate, some new secondary cracks would generate at the lower edge of the concrete which was gradually away from the mid-span of the RC beam. In stage II, the main crack propagated slowly and continuously, while other cracks stayed after their length reached to a certain level (50%~70% of RC beam’s height).
2. Initiation and propagation of interfacial crack. In stage II, interface stress of crack mouths increased due to stress concentration. When the interface stress was beyond the shear strength of the interface between CFL and concrete, a part debonding of CFL appeared, that is to say, the interfacial crack initiated. The interfacial crack propagated to one end of the strengthened beam gradually under cyclic ladder loads. After failure of the strengthened beam, the interface of the debonded CFL was shown in Figure 7b. As shown in this figure, a lot of concrete was pulled off from the beam. That is to say, the interface layer between CFL and concrete provided a good resistance to the interfacial crack propagation.
3. Yielding of main steel bars. In stage I and II, tension bars (main steel bars) provided a great resistance to the deformation of the specimen. In the same time, fatigue damage of the main steel bars accumulated gradually. At the end of stage II, the main steel bars yield because of their damage accumulation, and led to the interfacial crack propagation and a part debonding of CFL.
4. Failure of the strengthened beam. At the beginning of stage III, the deformation resistance was mainly provided by CFL-concrete interface, while main bars were already yielded. This led to acceleration of the interface cracks’ propagation and CFL local debonding. The local debonding of CFL aggravated the burden of the main bars and finally led to the main bars breaking, as shown in Figure 7c. After main bars’ fracture, interface crack propagated rapidly till complete debonding of one side of CFL, and concrete near the loading point was crushed, the strengthened beam was failed.

In conclusion, the fatigue failure mode of the RC beams strengthened with CFL under three-ladder spectrum can be considered as the partial debonding of CFL-concrete interface caused by the yield of the main steel bars, and finally led to the main steel bars fracture and the complete debonding failure of CFL.

## 5. Fatigue Life Analysis

### 5.1. Experimental $\bar{S_{R}}\sim N$ Curve

**Figure 8:**
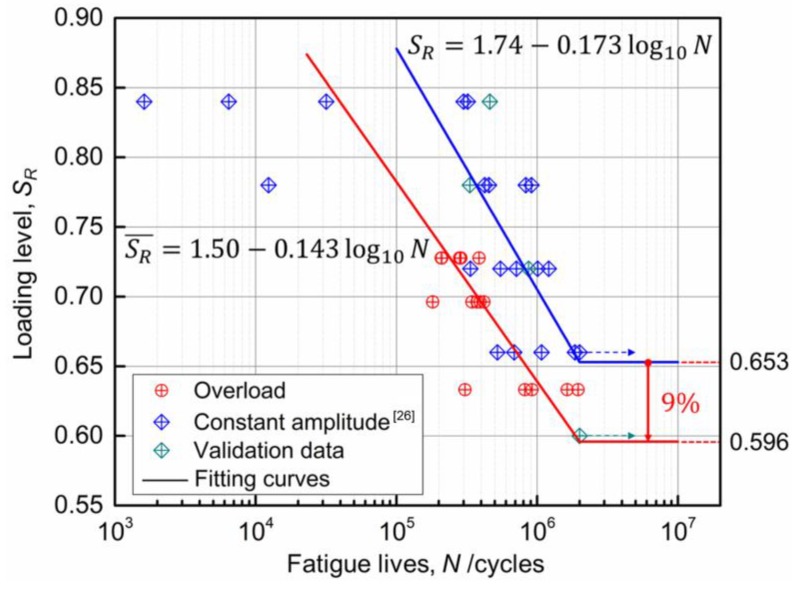
$\bar{S_{R}}\sim N$ and $S_{R}\sim N$ curve.

Based on the experimental method in Section 3.2, fatigue experiments of 15 RC beams strengthened with CFL were carried out under different loading levels of the three-ladder overload spectrum. Testing results are shown in Table 5. The loading levels of the three-ladder spectrum in Table 5 were calculated with weighted average method according to the loading cycles, and the weighted average loading level vs fatigue life curve of the strengthened beams, $\bar{S_{R}}\sim N$ curve was obtained. $\bar{S_{R}}\sim N$ curve and $S_{R}\sim N$ curve obtained by the fatigue tests with the same beams under constant amplitude cyclic loads [26] were shown in Figure 8. In addition, there are four validation beams under constant amplitude cyclic loads.

It can be seen in Figure 8, $\bar{S_{R}}\sim N$ curve was entirely under the $S_{R}\sim N$ curve. That is to say, when the loading level was the same, the fatigue lives of the strengthened beams under overload spectrum were much lower than that under constant amplitude cyclic loads. Moreover, the fatigue limit is reduced by 9% (0.596 under overload and 0.653 under constant amplitude cyclic load). Therefore, it would increase potential risk to carry out fatigue design of bridge structures under overload by using fatigue test results under constant amplitude loads.

To facilitate the fatigue design of the RC members strengthened with CFRP, a semi-empirical equation for the fatigue test curves of the strengthened beams under the overload ladder spectrum was proposed in this paper. Fatigue equations under corresponding load spectrum were obtained by least square fitting of the testing data under overload ladder spectrum and constant amplitude cyclic loads [26] in Figure 8.

Constant amplitude:

(4) $$S_{R}=1.74-0.173\log_{10}N,$$

Overload:

(5) $$\bar{S_{R}}=1.50-0.143\log_{10}N,$$

### 5.2. Fatigue Life Analysis

As mentioned above, the fatigue lives and fatigue limit of the strengthened beams under overload spectrum were much lower than that under constant amplitude cyclic loads. Therefore, constant amplitude fatigue results cannot be used directly in fatigue life prediction and fatigue design of the strengthened beams under overload spectrum. Fatigue cumulative damage theory was generally used for the fatigue life prediction of concrete members under variable amplitude loads. At present, Palmgren-Miner rule, modified Palmgren-Miner rule, Conten-Dolan damage theory are widely used in engineering, among which the Palmgren-Miner rule and modified Palmgren-Miner rule are widely used in engineering due to their simple form and easy calculation.

In this section, on one hand, Palmgren-Miner rule and modified Palmgren-Miner rule were used for predicting fatigue lives of the specimens under the overload ladder spectrum. On the other hand, an equivalent method was proposed to convert loading levels of the overload ladder spectrum into equivalent constant amplitude cyclic loading levels, based on the modified Palmgren-Miner rule with a semi-empirical fatigue equation of the strengthened beams under constant amplitude loads which established by this research group [26,27].

#### 5.2.1. Fatigue Life Prediction Based on Palmgren-Miner Rule

The overload ladder spectrum used in the fatigue tests was the repeat of 3 loading ladders. Without considering the effect of loading sequence, Palmgren-Miner rule could be used to preliminary predict the fatigue lives of the strengthened beams. Under variable amplitude loads, the ratio between loading frequency $n_{i}$ of constant amplitude load $S_{i}$ and fatigue life of the strengthened beams N was:

(6) $$\lambda_{i}=\frac{n_{i}}{N},$$

According to Palmgren-Miner rule, the damage D was:

(7) $$D=\sum \frac{n_{i}}{N_{i}}=\sum \frac{\lambda_{i}N}{N_{i}}=N\sum \frac{\lambda_{i}}{N_{i}}=1,$$

where $N_{i}$ is the fatigue lives under constant amplitude load $S_{i}$.

According to the preliminary study result obtained by this research group [27], $S_{i}∼N_{i}$ curve equation can be expressed as:

(8) $$S_{i}=A+B\log_{10}N_{i},$$

From Equation (6) to (8), it can be concluded that:

(9) $$N=\frac{D}{\sum \frac{\lambda_{i}}{10^{\frac{S_{i}-A}{B}}}}=\frac{D}{\sum \lambda_{i}\times 10^{-\frac{S_{i}-A}{B}}},$$

On the premise that the S∼N curve equation of the strengthened beams under constant amplitude loads was known, Equation (9) can be used to predict the fatigue lives of the strengthened beams under variable amplitude loads.

In case of the RC beams strengthened with CFL under the three-ladder overload spectrum, the fatigue lives $N_{pm}$ can be predicted by Equations (9) and (4), and the critical damage values $D_{c}$ with the linear damage accumulation rule at the time of specimen failure were calculated, as shown in Table 6.

It can be seen that there were large relative errors in life predicting results based on Palmgren-Miner rule, and all of the predicted values were higher than average test data. Therefore, it is infeasible to predict fatigue lives of the RC beams strengthened with CFL under overload ladder spectrum with linear accumulation Palmgren-Miner rule.

#### 5.2.2. Fatigue Life Prediction Based on Modified Palmgren-Miner Rule

The average value of $D_{c}$ in Table 6 was:

(10) $$\bar{D_{c}}=0.522,$$

Taking $q=\bar{D_{c}}$ as the correction coefficient, a modified Palmgren-Miner rule can be established as follows:

(11) $$D=\sum \frac{n_{i}}{q\cdot N_{i}}=\sum \frac{\lambda_{i}N}{q\cdot N_{i}}=N\sum \frac{\lambda_{i}}{q\cdot N_{i}}=1,$$

From Equations (8) and (11), the fatigue lives of the strengthened beams under three-ladder overload spectrum can be calculated by:

(12) $$N=\frac{q\cdot D}{\sum \frac{\lambda_{i}}{10^{\frac{S_{i}-A}{B}}}}=\frac{q\cdot D}{\sum \lambda_{i}\times 10^{-\frac{S_{i}-A}{B}}},$$

Using Equation (12) and the semi-empirical fatigue Equation (4), the fatigue lives of the strengthened beams of group A1~A3 were predicted, and the results are shown in Table 7. As shown in Table 7, the prediction of the fatigue lives of the RC beams strengthened with CFL under the overload ladder spectrum had a high accuracy, and the average relative error was 18.2%. This showed that the prediction method was effective and feasible.

#### 5.2.3. Loading Level Equivalent Method Based on Modified Palmgren-Miner Rule

Based on the modified Palmgren-Miner rule mentioned above, and damage equivalence method, loading levels of three ladders in the overload spectrum were converted into an equivalent loading level of constant amplitude cyclic loads. The calculating formula was:

(13) $$S_{e}={\left(\frac{\sum n_{i}S_{i}^{m}}{q\cdot N}\right)}^{1/m},$$

where $S_{e}$ is the equivalent loading level of constant amplitude cyclic loads, m=8.19 is fatigue strength coefficient.

Using Equations (13), (12) and (4), loading levels of group A1~A3 were converted, as shown in Table 8.

According to equivalent loading level in Table 8 and testing data in Table 5, the equivalent loading level vs. fatigue life ($S_{e}\sim N$) curve was obtained, and the fatigue equation was:

(14) $$S_{e}=1.64-0.158\log_{10}N,$$

**Figure 9:**
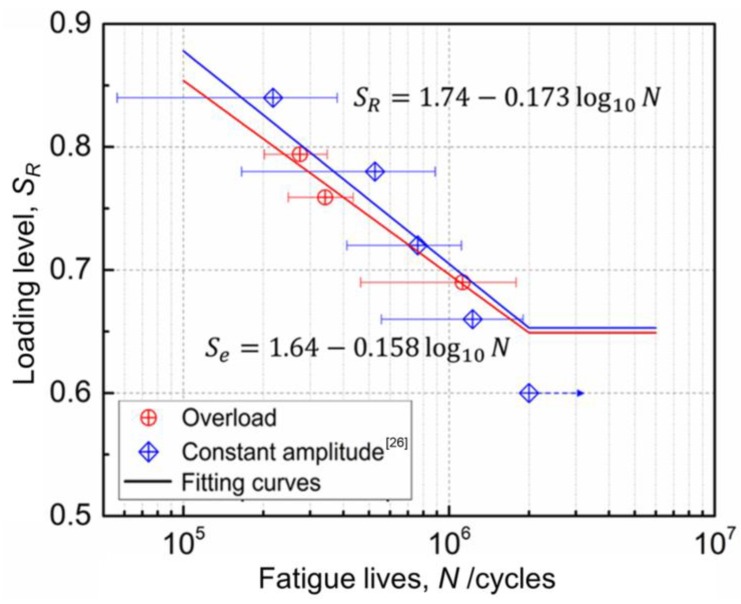
$S_{e}\sim N$ and $S_{R}\sim N$ curve.

The $S_{e}\sim N$ curve was compared to $S_{R}\sim N$ curve of the specimens under constant amplitude cyclic loads, as shown in Figure 9. It can be seen that the $S_{e}\sim N$ curve was closed to the $S_{R}\sim N$ curve. That is to say, when the equivalent loading level was the same, the fatigue life of the strengthened beams under overload ladder spectrum was shorter than that under constant amplitude cyclic loads, but the relative errors were smaller (the average relative error was 20.8%). Moreover, their fatigue limits were close (0.649 under overload and 0.653 under constant amplitude cyclic load) and the relative error was only 0.61%. Therefore, it is effective and feasible to use the load level equivalent method based on the modified Palmgren-Miner rule to predict the fatigue lives of the strengthened beams and establish $S_{e}\sim N$ curve. Using this method, the anti-fatigue design of the strengthened beams under overload can be implemented.

## 6. Conclusions

In this paper, RC member strengthened with CFRP of highway bridge was taken as the research object. In view of the actual situation of vehicle overload in China, the fatigue experimental method of RC members strengthened with CFRP under vehicle overload was proposed. The fatigue performance of RC beams strengthened with CFRP under the overload ladder spectrum was discussed. The following conclusions were obtained:

1. Based on the traffic data of a highway in Guangzhou collected by this research group in the earlier period, a definition method of vehicle overload was proposed. According to the typical bridge structure, the overload level of the structure under vehicle living load was calculated, and a three-ladder vehicle overload spectrum was compiled.
2. Based on the above vehicle overload ladder spectrum, the fatigue experimental method of RC members strengthened with CFRP under the overload ladder spectrum was proposed, and fatigue experiments of RC beams strengthened with CFL under the overload ladder spectrum with three loading levels were carried out successfully.
3. The fatigue failure mechanism analysis and experimental results of RC beams strengthened by CFL show that, under the same equivalent loading level, the fatigue lives of the strengthened beams under overload ladder spectrum were lower than that under constant amplitude cyclic loads, and the fatigue limit was reduced by 9%. Therefore, for the bridge components subjected to overload, if the fatigue test data under constant amplitude cyclic loads is directly used for anti-fatigue design, it would bring safety risk to the bridge.
4. A modified Palmgren-Miner rule was established, and the fatigue life prediction methods based on the modified Palmgren-Miner rule and load level equivalent method were proposed. The results of fatigue life analysis of RC beams strengthened with CFL show that, it is effective and feasible to apply the life prediction methods based on the modified Palmgren-Miner rule and load level equivalent method to carry out the anti-fatigue design of RC structures strengthened with CFRP under overload.

## Figures and Tables

**Table 1:** Equivalent moments and overload levels.

Overload Intervals/kN·m	$M_{ve}/kN\cdot m$	$M_{vo}/kN\cdot m$	Overload Level $(M_{vo}+M_{1})/M_{u}$	Vehicle Frequencies $N_{v}$/Cycles
[800, 1200]	1052	577	0.623	535
(1200, 1400)	1302	715	0.682	81
[1400, 2000)	1506	828	0.732	15

**Table 2:** Main parameters of the testing spectrum.

Ladder	$P_{max}/kN$	$P_{min}/kN$	Stress Ratio R	Ladder Length/Cycles
I	28.0	2.80	0.1	535
II	30.7	3.07	0.1	81
III	32.9	3.29	0.1	15

**Table 3:** Mechanical properties of concrete.

Cube Compressive Strength	Elastic Modulus	Axial Compressive Strength	Flexural Strength	Flexural Modulus	Poisson’s Ratio
45.6 MPa	31.2 GPa	28.9 MPa	4.25 MPa	28 GPa	0.193

**Table 4:** Basic mechanical properties of CFL.

Tensile Strength	Elastic Modulus	Computing Thickness	Elongation Rate
4750 MPa	230 GPa	0.23 mm	1.5%

**Table 5:** Experimental conditions and results.

Group No.	Specimen No.	Load Ladders	Peak Load $P_{max}/\mathrm{kN}$	Loading Levels $S_{R}$	Ladder Length $l_{i}/\mathrm{Cycles}$	Fatigue Lives $N_{f}/\mathrm{Cycles}$
A1	A11	I	28.0	0.623	535	1,633,260
	A12					1,950,810
	A13	II	30.7	0.682	81	816,502
	A14					307,110
	A15	III	32.9	0.731	15	919,355
A2	A21	I	30.8	0.684	535	392,417
	A22					181,003
	A23	II	33.8	0.751	81	374,291
	A24					343,815
	A25	III	36.2	0.804	15	417,781
A3	A31	I	32.2	0.716	535	287,064
	A32					281,428
	A33	II	35.3	0.784	81	386,159
	A34					208,084
	A35	III	37.8	0.840	15	210,078

**Table 6:** Fatigue life predicting and critical damage calculating results based on Palmgren-Miner rule.

Group No.	Loading Levels $S_{R}$	Average Test Data N/Cycles	Predicted Lives $N_{pm}/\mathrm{Cycles}$	Relative Errors/%	Critical Damages $D_{c}$
A1	0.623, 0.682, 0.731	1,125,407	1,691,598	50.3	0.665
A2	0.684, 0.751, 0.804	341,861	817,801	139	0.418
A3	0.716, 0.784, 0.840	274,563	568,541	107	0.483

**Table 7:** Fatigue life predicting results by Equation (12).

Group No.	Loading Levels $S_{R}$	Average Test Data N/Cycles	Predicted Lives $N_{pm-c}/\mathrm{Cycles}$	Relative Errors/%
A1	0.623, 0.682, 0.731	1,125,407	883,151	−21.5
A2	0.684, 0.751, 0.804	341,861	426,958	24.9
A3	0.716, 0.784, 0.840	274,563	296,824	8.11

**Table 8:** Calculating results for equivalent loading levels.

Group No.	Overload Ladder	Loading Levels $S_{R}$	Equivalent Loading Levels $S_{e}$
A1	I	0.623, 0.682, 0.731	0.690
A2	II	0.684, 0.751, 0.804	0.759
A3	III	0.716, 0.784, 0.840	0.794
